# Mosaic somatic *HRAS* mutation causes unilateral psoriasis

**DOI:** 10.1093/lifemedi/lnad018

**Published:** 2023-05-10

**Authors:** Rina Su, Huanwei Huang, Ying Chang, Yi Yu, Jun Cui, Ting Chen, Bin Li, Wenhui Wang

**Affiliations:** Department of Dermatology, Beijing Chao-yang Hospital, Capital Medical University, Beijing 100020, China; National Institute of Biological Sciences, Beijing 102206, China; School of Basic Medical Sciences, Capital Medical University, Beijing 100069, China; National Institute of Biological Sciences, Beijing 102206, China; National Institute of Biological Sciences, Beijing 102206, China; National Institute of Biological Sciences, Beijing 102206, China; National Institute of Biological Sciences, Beijing 102206, China; Tsinghua Institute of Multidisciplinary Biomedical Research, Tsinghua University, Beijing 100084, China; National Institute of Biological Sciences, Beijing 102206, China; Department of Dermatology, Peking University Third Hospital, Beijing 100191, China

Dear Editor,

Psoriasis is a common inflammatory skin disease that can be caused by a variety of complex intrinsic and extrinsic factors. Previous studies have generated a well-accepted paradigm underneath the psoriasis pathology in the skin. Immunological and genetic studies revealed that IL-23-mediated activation of the Th17 pathway is the key driver of psoriasis phenotype in patients’ skin. In addition, crosstalk among keratinocytes, dendritic cells, and IL-17-producing cells (e.g., Th17) plays essential roles during the progression of psoriasis. On the other hand, the upstream causes of psoriasis are less clear. Genetic factors are considered to be the main risk determinants for the initiation and development of psoriasis, while environmental factors can also be involved in triggering and exacerbating psoriasis in patients [1]. Genetic analysis of monogenic psoriasis cases and genome-wide association studies can provide important knowledge into the initiation mechanism of psoriasis. But, often, the reported genetic variants lack genetic studies using disease models to gain causal and mechanistic insights.

Previous studies revealed that increased expression of *HRAS* and enhanced GTP-binding activity of Ras can be observed in psoriasis patients’ skin [2, 3]. Despite the mounting evidence linking *HRAS* activity and psoriasis pathogenesis, there has been no reported case of human psoriasis associated with *HRAS* mutations to date.

Unilateral psoriatic lesions occur mostly along the so-called Blaschko lines in a unilateral fashion, and its mechanism is largely unknown, although genetic mosaicism has been proposed to be the underlying cause. Elucidating the genetic driver for unilateral psoriasis can provide a unique angle into understanding the initiation mechanism and genetic susceptibility of psoriasis because it eliminates interpersonal variations in genetic background, external environment stimuli, systemic diseases, and other factors.

We studied a 22-year-old Chinese male with recurrent linear red plaques with scaling and itching affecting only the left side of his body for 3 years (Fig. 1A). One of his uncles was also diagnosed with psoriasis. Triggers that aggravated the patient’s skin symptoms included psychological stress, spicy food, and seasonal flu. The patient showed no joint, nail, palmoplantar, or vulvar involvement. The patient’s skin lesions could be cleared after treatment with topical steroids but recurred after therapy discontinuation. Spontaneous resolution occurred sometimes. Physical examination demonstrated demarcated scaly erythematous papules and plaques along Blaschko’s lines on the left trunk, buttocks, and extremities. Psoriasis-specific “wax spot phenomenon,” “surface membrane phenomenon,” and “pinpoint bleeding (Auspitz sign)” were positive. No sebaceous nevus or epidermal nevus were observed. The patient has been diagnosed with unilateral psoriasis by three separate board-certified dermatologists based on classic findings of skin lesions and disease history combined with family history.

**Figure 1.**
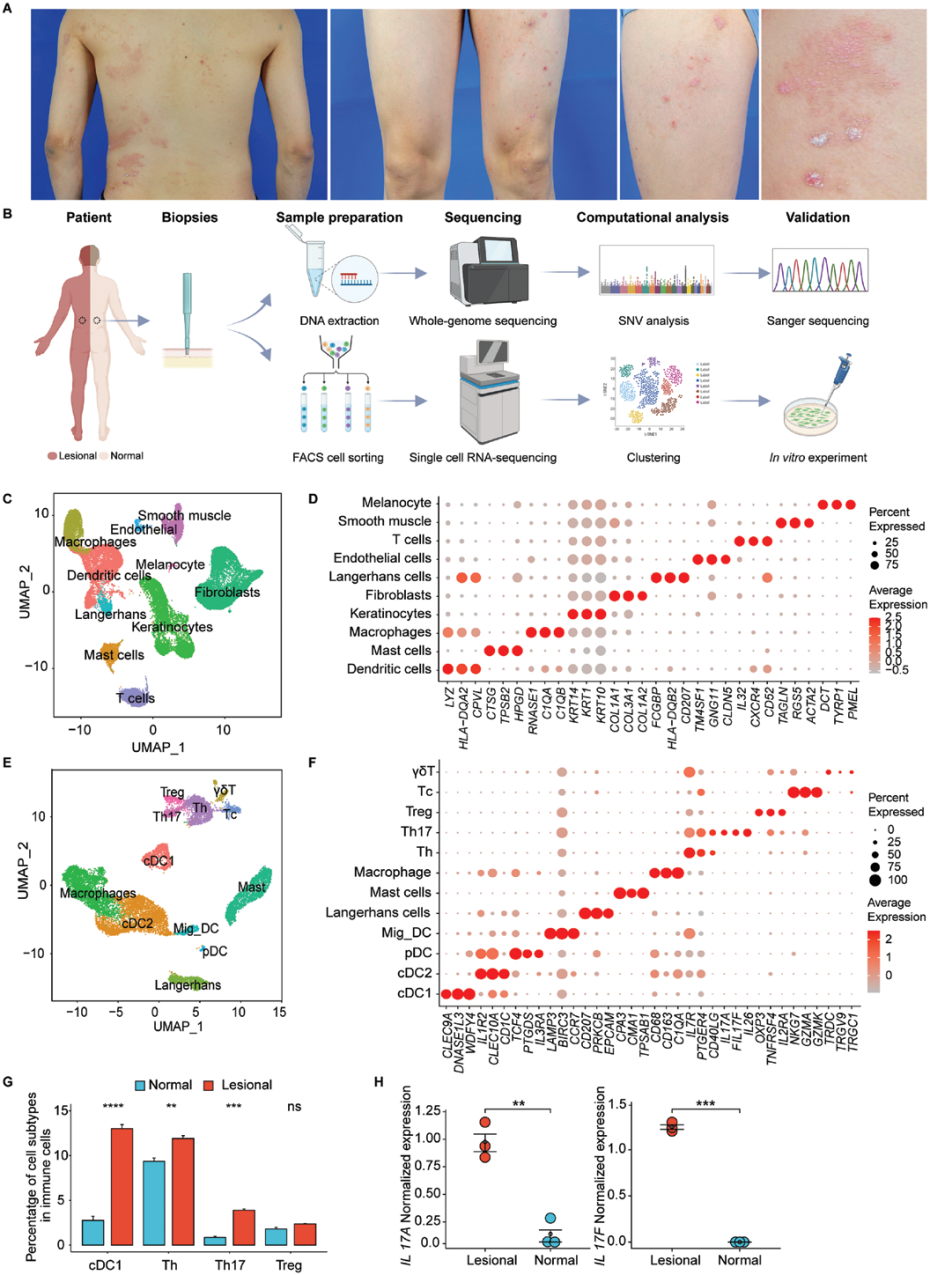
Single-cell RNA-seq analysis of a patient with unilateral psoriasis. (A) Images of skin rashes on left trunk, arm, and leg of the patient. (B) Workflow of biopsies, sample preparation, sequencing, computational analysis of scRNA-seq data and whole-genome sequencing (WGS) data, and *in vitro* validations. Created with BioRender.com. (C, E) UMAP visualizations of the integrated cell embeddings for scRNA-seq data, including 37,138 cells from the entire dataset (C) and 14,157 cells from the immune cell subset (E). Each dot represented a single cell colored by cell type annotations. (D, F) Expressions of specific marker genes in each cell type and immune cell subtypes, in which the top 3 signature genes for each cell type are showed. (G) Percentage of cDC1, Th, Th17, and Treg cells in immune cells from the normal and lesional samples (two-sided Wilcoxon Sum Rank test, Bonferroni correction). **P-value < 0.01; ***P-value < 0.001; ****P-value < 0.0001. (H) Expression of the *IL17A* and *IL17F* genes in the normal and lesional samples (two-sided Wilcoxon Sum Rank test, Bonferroni correction).

To understand the skin pathology at the cellular level, we used scRNA-seq to analyze all cell types present in the lesional side of the skin with pathological symptoms, as a control we used cells isolated from the non-lesional side of the skin from the same patient (Fig. 1B). Freshly obtained skin biopsies taken from symmetric locations of the same patient’s back skin were enzymatically dissociated to isolate single cells for FACS sorting. FACS-isolated cells from each biopsy were divided into three replicated samples and submitted for sequencing analysis (Table S1). Unsupervised clustering of a total of 37,138 cells from 6 experimental batches yielded 10 major cell types based on the expression of canonical marker genes, including keratinocytes (*KRT14*, *KRT1*, and *KRT10*), fibroblasts (*COL1A1*, *COL3A1*, and *COL1A2*), dendritic cells (*LYZ, HLA-DQA2,* and *CPVL*), T cells (*IL-32, CXCR4,* and *CD52*), macrophages (*RNASE1, C1QA,* and *C1QB*), Langerhans cells (*CD207, HLA-DQB2,* and *FCGBP*), smooth muscle cells (*ACTA2, TAGLN,* and *RGS5*), mast cells (*CTSG, TPSB2,* and *HPGD*), endothelial cells (*TM4SF1, GNG11,* and *CLDN5*), and melanocytes (*DCT, TYRP1,* and *PMEL*) (Fig. 1C and 1D).

Among the cell types identified in the skin, we further analyzed the transcriptional profiles of immune cells, the major cell type responsible for inflammatory symptoms in psoriasis skin. We identified 12 cell subtypes for the immune cells, including conventional type 1 dendritic cells (cDC1) (*CLEC9A, DNASE1L3,* and *WDFY4)*, conventional type 2 dendritic cells (cDC2) (*CLEC10A, IL1R2,* and *CD1C*), plasmacytoid dendritic cells (pDC) (*TCF4, PTGDS*, and *IL3RA*), migratory dendritic cells (Mig_DC) (*LAMP3, BIRC3,* and *CCR7*), Langerhans cells (*CD207,  PRKCB,* and *EPCAM*), mast cells (*CPA3, CMA1,* and *TPSAB1*), macrophages (*CD68, CD163,* and *C1QA*), T helper cells (Th) (*IL7R, PTGER4,* and *CD40LG*), *IL17*^+^ T helper cells (Th17) (*IL17A, IL17F,* and *IL26*), regulatory T cells (Treg) (*FOXP3, TNFRSF4,* and *IL2RA*), cytotoxic T cells (Tc) (*NKG7, GZMA,* and *GZMK*), and γδT cells (*TRDC, TRGV9,* and *TRGC1*) (Fig. 1E and 1F). Quantification of the percentage of Th17 cells and the expression levels of *IL17A/F* using scRNA-seq data showed they were significantly higher in the lesional skin than in the non-lesional skin (Fig. 1G and 1H), both of which are considered as hallmarks in the pathogenesis of psoriasis [1]. In addition, the lesional side had increased percentages of cDC1 and Th cells compared to the non-lesional side, implying enhanced antigen presentation, cytokine production, and activation of immune responses (Fig. 1G). Overall, these results showed that the transcriptomic profile of the patient was consistent with the conventional psoriasis transcriptome, validating the clinical diagnosis of unilateral psoriasis.

To investigate whether or not somatic mosaic mutations caused the unilateral psoriasis, we performed whole-genome sequencing using genomic DNA isolated from the lesional side of the patient skin; as a control, we used genomic DNA isolated from the non-lesional side of the skin from the same patient (Fig. 1B; Table S2). We identified different genomic variations that are uniquely presented in the lesional sample, including single-nucleotide variations (SNVs), deletions, and insertions. These variations are distributed throughout the genome, ranging from exons, introns, untranslated regions (UTRs) to intergenic regions on different chromosomes. Since exons contain 85% of disease-driving mutations and disease-predisposing single-nucleotide polymorphisms, we next focused on the only three nonsynonymous SNVs in the exon regions, including *ALG9*^*L45C*^, *RSBN1*^*K93T*^, and *HRAS*^*G12S*^ (Fig. 2A). The somatic missense *HRAS*^*G12S*^ variant had the highest allele frequency of 17.8% among all three candidate SNVs (Fig. 2B). The presence of the G12S somatic mutation in affected skin was further validated by allele-specific PCR and Sanger sequencing of PCR products (Fig. 2C). However, *ALG9*^*L45C*^ and *RSBN1*^*K93T*^ cannot be validated using Sanger sequencing of PCR products.

**Figure 2.**
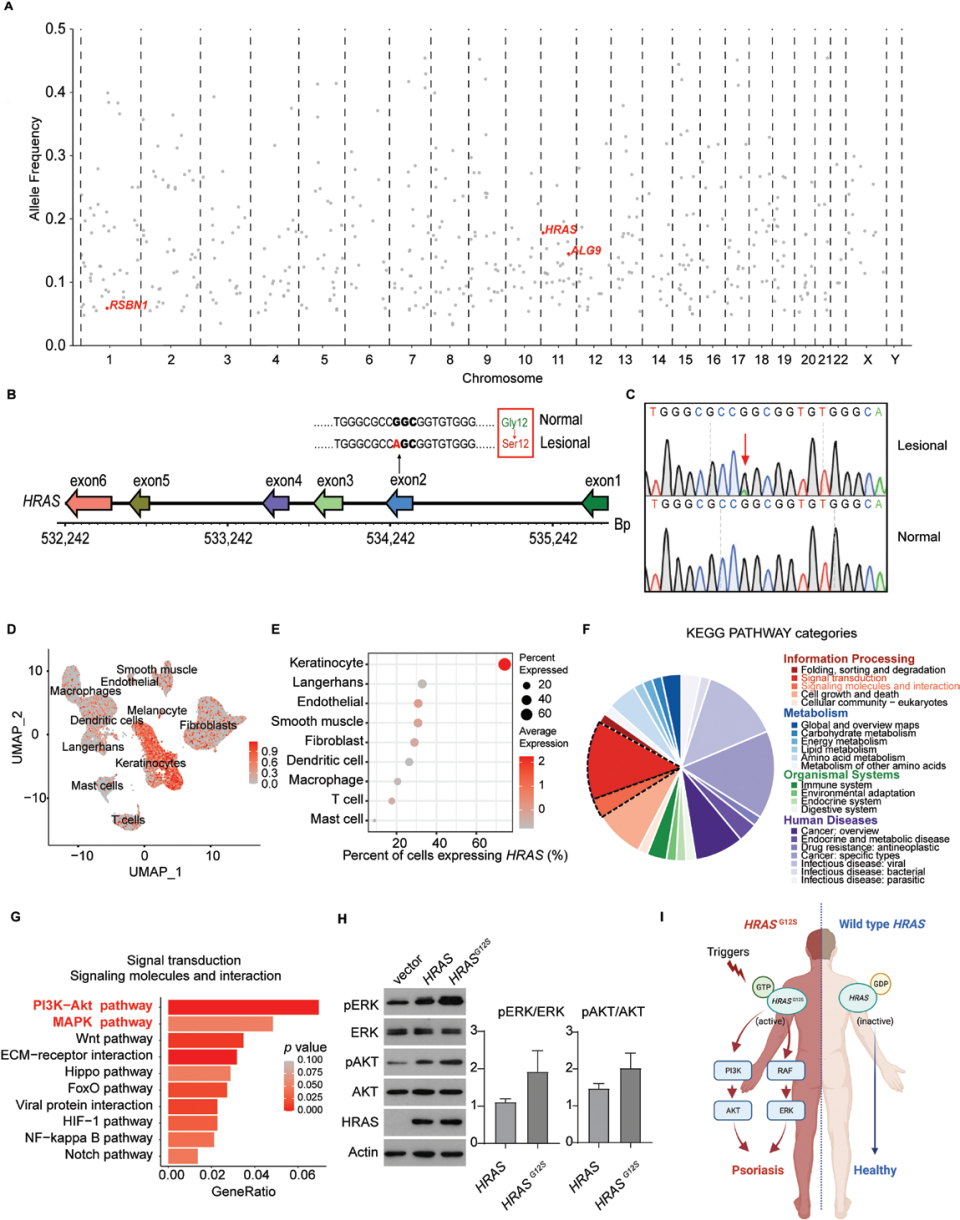
WGS analysis reveals mosaic somatic *HRAS* mutation in lesional skin. (A) Dot plots showed the genetic variants in the whole genome located in different chromosomes, where *Y* axis showed the allele frequency of each variant. Red dots represented nonsynonymous variants in exon region. (B) The wild type and mutated sequences of the second exon of *HRAS* gene show a G > S single-nucleotide polymorphism at the 12th base pair, i.e., the *HRAS*^G12S^ mutation. (C) Sanger sequencing of the DNA fragment around the *HRAS*^G12S^ mutation. (D) Expression of *HRAS* in the cells of the scRNA-seq data. (E) Percentage of cells in which the expression of *HRAS* was detected in each cell type. (F) KEGG pathways significantly enriched in keratinocytes were classified into four main categories and 22 subcategories. (G) KEGG pathway analysis results of genes highly expressed in keratinocytes from the lesional samples compared with those from the normal samples. (H) Western blotting showed that the *HRAS*^G12S^ mutation lead to enhanced phosphorylation of ERK and AKT in HaCaT cells. Band intensities were normalized to that of the non-phosphorylated protein. Bar heights, mean values; whiskers, standard deviations; *n* = 3. (I) Schematic models for the function of *HRAS*^G12S^ mutation in an unilateral psoriasis patient.

*HRAS* is a well-studied proto-oncogene. The G12 mutation prevents the intrinsic GAP-catalyzed hydrolysis of GTP, resulting in persistent activation of RAS molecules and activation of the MAPK and PI3K-Akt signaling pathways [4]. To the best of our knowledge, this patient represents the first reported case of unilateral psoriasis with somatic *HRAS*^G12S^ mosaic mutation.

Next, we examined the expression pattern of *HRAS* gene in the scRNA-seq data of the patient and found that *HRAS* was mainly expressed in keratinocytes (Fig. 2D and 2E). Functional annotation analysis of keratinocytes revealed that PI3K−Akt signaling pathway and MAPK signaling pathway were significantly enriched in the lesional side than that in the non-lesional side skin (Fig. 2F and 2G). To further validate the causal relationship between *HRAS*^*G12S*^ mutation and PI3K-Akt and MAPK signaling pathways, we infected HaCaT cells, an immortalized human keratinocyte cell line, with *HRAS*, *HRAS*^*G12S*^, and empty vector using lentiviruses. Consistent with previous studies [5], overexpression of *HRAS*^G12S^ enhanced ERK and AKT phosphorylation in HaCaT cells (Fig. 2H), indicating the PI3K-Akt and MAPK signaling pathways were activated by mutant *HRAS*^*G12S*^.

Here by systemic analysis of a unique unilateral psoriasis patient, we identified a gain-of-function variant of *HRAS*^*G12S*^ only found in the lesional side of the patient skin using whole-genome sequencing. Based on scRNA-seq analysis of skin samples isolated from the lesional side and non-lesional side of the same patient, we found enhanced PI3K-Akt and MAPK signaling pathway in keratinocytes in the lesional side of the skin. Since *HRAS* expression is enriched in keratinocytes based on our scRNA-seq analysis, we carried out *in vitro* cell culture experiments using human keratinocytes cell line and validated that *HRAS*^*G12S*^ enhanced PI3K-Akt and MAPK signaling (Fig. 2I).

Somatic *HRAS* mutation has been reported to be associated with epidermal nevi and nevus sebaceous [6, 7]. Although the inflammatory linear verrucous epidermal nevus might resemble linear psoriasis clinically and histologically, the entity is significantly different from psoriasis. In our patient, the complete remission and relapsing course, lesions scatter and vary in size, adulthood onset, environmental triggers, family history of psoriasis, significantly higher levels of Th17 cells and *IL17A/F* in the lesional skin, are all in favor of the diagnosis of psoriasis and against epidermal nevus or sebaceous nevus.

Previously published mouse genetic study showed that the expression of constitutively activated *Hras* in keratinocytes of adult mice developed a psoriasis-like skin phenotype, demonstrating the ability of activated *Hras* to initiate skin inflammation, keratinocyte proliferation and activation of skin Th17 cells [8]. In addition, epidermal expression of *Raf*, one of the downstream effectors of Ras GTPases, caused epidermal hyperplasia and the neutrophil-dominant cutaneous inflammatory reactions which are characteristics of psoriasis in mice [9]. Coupled with these mouse genetic studies, our finding here provided the first reported case of human psoriasis caused by a somatic mosaic gain-of-function mutation in *HRAS*.

Our scRNA-seq analysis and *in vitro* cell culture study revealed that PI3K−Akt and MAPK signalling pathways were activated in the lesional skin keratinocytes of the unilateral psoriasis patient. These results imply that suppression of RAS signaling or targeting *HRAS*^G12S^ mutation could be an option for the treatment of psoriasis in the future, especially for patients harboring *HRAS* mutations. Currently, several potential therapeutic molecules targeting RAS signaling pathway are undergoing preclinical or clinical trials for the treatment of human cancers [4]. Several studies have reported remarkable therapeutic improvement of chronic psoriasis in cancer patients during the treatment of EGFR inhibitors, which can inhibit RAS signaling pathway [10]. Our genetic and scRNA-seq analysis provided a solid basis for using these targeted approaches to treat human psoriasis in the future.

## Research limitations

Due to the rare nature of the unilateral psoriasis cases, this study only provided an analysis of one patient. More clinical samples should be used to study the prevalence and frequency of *HRAS* mutations in general psoriasis patients in the future.

Another potential limitation is that the diagnosis of psoriasis could be further complemented by traditional histopathology analysis. We obtained the patient’s consent to acquire skin biopsy based on the principle of minimizing skin trauma associated with the invasive examinations. Even though we believe the diagnosis of unilateral psoriasis based on clinical features and RNA transcriptional profiles surpassed traditional section staining analysis of patient skin.

## Supplementary Material

lnad018_suppl_Supplementary_Data
