# Supplementary material for: Mosaic somatic HRAS mutation causes unilateral psoriasis
Source: Life Med. 2023 May 10;2(3):lnad018. doi: 10.1093/lifemedi/lnad018 (PMC11748982; doi:10.1093/lifemedi/lnad018)
Supplement: lnad018_suppl_Supplementary_Data [file lnad018_suppl_Supplementary_Data.docx]

**Supplementary Information for “Mosaic somatic HRAS mutation causes unilateral psoriasis”**

Table S1. Quality control of single cell-RNA seq data.

|  | CN09-1 | CN09-2 | CN09-3 | PS04-1 | PS04-2 | PS04-3 |
| --- | --- | --- | --- | --- | --- | --- |
| Estimated Number of Cells | 5,377 | 5,601 | 5,376 | 18,385 | 17,337 | 16,200 |
| Mean Reads per Cell | 204,706 | 153,114 | 167,350 | 73,781 | 55,152 | 65,050 |
| Median Genes per Cell | 2,317 | 2,257 | 2,356 | 1,256 | 1,484 | 1,501 |
| Number of Reads | 1,100,704,368 | 857,593,457 | 899,675,495 | 1,356,459,410 | 956,177,963 | 1,053,807,721 |
| Valid Barcodes | 97.50% | 97.40% | 97.30% | 97.50% | 97.50% | 97.40% |
| Sequencing Saturation | 86.00% | 82.60% | 83.10% | 72.00% | 64.80% | 69.30% |
| Q30 Bases in Barcode | 96.00% | 94.90% | 95.00% | 96.00% | 95.50% | 95.60% |
| Q30 Bases in RNA Read | 92.90% | 87.90% | 87.60% | 91.50% | 90.80% | 89.60% |
| Q30 Bases in UMI | 93.90% | 93.50% | 93.70% | 94.10% | 94.40% | 94.40% |
| Reads Mapped to Genome | 96.70% | 95.40% | 95.10% | 96.50% | 96.60% | 96.30% |
| Reads Mapped Confidently to Genome | 82.10% | 81.80% | 81.00% | 86.50% | 87.20% | 86.60% |
| Reads Mapped Confidently to Intergenic Regions | 3.90% | 4.30% | 4.20% | 4.10% | 4.20% | 4.40% |
| Reads Mapped Confidently to Intronic Regions | 18.20% | 18.80% | 18.70% | 18.30% | 18.40% | 18.80% |
| Reads Mapped Confidently to Exonic Regions | 60.00% | 58.70% | 58.10% | 64.00% | 64.50% | 63.40% |
| Reads Mapped Confidently to Transcriptome | 56.80% | 55.50% | 54.90% | 60.50% | 61.00% | 59.90% |
| Reads Mapped Antisense to Gene | 1.50% | 1.50% | 1.60% | 1.60% | 1.60% | 1.60% |
| Fraction Reads in Cells | 79.50% | 80.10% | 79.70% | 76.60% | 75.80% | 75.80% |
| Total Genes Detected | 25,992 | 25,987 | 26,018 | 28,415 | 27,973 | 27,930 |
| Median UMI Counts per Cell | 7,800 | 7,356 | 8,014 | 3,389 | 3,875 | 3,956 |
| Number of Doublet Cells | 124 | 161 | 158 | 706 | 726 | 594 |
| Number of Filtered Cells | 3864 | 3961 | 3894 | 8584 | 8821 | 8014 |

Table S2. Quality control of single cell-RNA seq data.

|  | total read count | mapped reads | average depth | coverage (%) |
| --- | --- | --- | --- | --- |
| L-Normal | 1560886154 | 1560886154 | 72.47 | 88.66 |
| K-R-N | 1206458337 | 1206458337 | 57.36 | 88.67 |
| R-Normal | 1596999977 | 1596999977 | 74.21 | 88.71 |
| K-L-L | 1479224911 | 1479224911 | 69.71 | 88.74 |

**METHODS DETAILS**

**Ethics statement**

The whole genomic DNA sequencing and scRNA-seq of human skin biopsies study were reviewed and approved by the Medical Science Research Ethics Committee of Peking University Third Hospital. The donor signed the written informed consent for sample collection and data analysis. Before signing the consent, necessary information including the goals and related experimental procedures for the study were provided to the donor. In this study, we collected 1 × 1 cm skin biopsies from the psoriasis lesion and contralateral control skin.

**Isolation of cells and Fluorescence-activated Cell Sorting**

For the isolation of single-cell suspensions using human skin biopsies, subcutaneous fat was removed from skins with a scalpel. Back skin was cut into pieces and placed dermis side down in 2.4 U/mL dispase II (Gibco, 17105041)) dissolved in PBS for 1 hr at 37˚C on an orbital shaker at 80 rpm. Separate the epidermis from the dermis with fine forceps and float the epidermis on 0.25% trypsin solution at 37˚C with shaking at 80 rpm for 10 min, and afterward 5% FBS medium was added to neutralize trypsin. Put the dermis in collagenase I (Sigma-Aldrich, C2674) at 37 °C with shaking at 80 rpm for 1 hr, afterward 5% FBS medium was added.

Single-cell suspensions from the epidermis or dermis were obtained by pipetting the tissue suspension up and down using 50 mL serological pipets. Cells were then filtered with strainers (70 μm followed by 40 μm), centrifuged, and stained with anti-human CD45 (BD Biosciences, 555485). DAPI staining was used to exclude dead cells.

On the basis of FACS analysis, single cells of different subtypes, including immune cells (CD45+) from both the epidermis and dermis, epidermal cells (CD45-) from the epidermis, dermal cells (CD45−) from the dermis were sorted into 2% BSA (Takara, 2320) in PBS. Cell analysis and isolation were performed on a BD Arial III cell sorter controlled by FACSDiva software (BD bioscience). For each skin biopsy sample, immune cells, dermal cells, and epidermal cells were pooled together in a 4:5:1 ratio for the next step of single-cell analysis.

**Isolation of Genomic DNA**

Genomic DNA was extracted from human skin biopsies with a genomic DNA isolation kit (Omega) according to the manufacturer’s instructions. Genomic DNA was measured with a NanoDrop instrument (Thermo Fisher Scientific).

**Single cell RNA-seq analysis**

Single-cell sequencing data were processed using the CellRanger pipeline (version 6.0.2; 10X Genomics). We mapped the raw data of each sample against the human genome reference (GRCh38), and annotated them with GENCODE gene annotations for the GRCh38 genome assembly (GENCODE release 32) to obtain expression matrix.

The single cell expression matrices for the 6 samples were then imported to R (4.1.0) using the Read10X() function from the Seurat package (4.0.5) (Hao et al., 2021). Cells with <500 genes or <2000 unique molecular identifiers (UMIs), and genes expressed in fewer than 200 cells were excluded from the dataset. Cells with over 25% mitochondrial gene expressed were also discarded. Then we normalized the expression matrix of each sample using the LogNormalize() function of Seurat with a scale factor = 10,000, and applied the Scale.Data() function of Seurat with “vars.to.regress” parameter to scale the read count of each cell. We selected top 2000 genes with highest dispersions using the FindVariableGenes() function. Moreover, we applied DoubletFinder (v2.0.3) with parameters “PCs = 1:30, pN = 0.25” to filter doublets from these expression matrices (McGinnis et al., 2019).

We used the FindIntegrationArchor() and IntegrateData() functions of Seurat to integrate the expression matrices for the 6 samples, and applied the FindClusters() function with “resolution = 0.5” to cluster cells of the integrated dataset. All cell clusters were then visualized in a 2-dimensional UMAP plot, and we annotated these clusters to known cell types using corresponding marker genes.

We extracted Dendritic cells, Langerhans cells, Macrophages, Mast cells, and T cells from the integrated dataset as an immune cell subset, including 14157 cells. We then re-clustered the immune cell subset using top 50 principal components and “resolution=1.5”. Differentially expressed genes for each immune cell cluster were generated by the FindAllMarkers() function and two-sided non-parametric Wilcoxon rank sum test (log(fold-change)>0.1, adjusted P-value<0.01).

We used the clusterProfiler package (version 4.0.2)(Wu et al., 2021) to determine the GO terms and KEGG pathways enriched for the differentially expressed genes of each cell cluster. To identify cell-type-specific biological signatures associated with skin lesion, we first categorized all KEGG pathways significantly enriched in keratinocyte to 22 subcategories according to the definition in the GenomeNet Database (https://www.genome.jp/kegg/pathway.html). We then clustered these 22 subcategories to four main categories based on their biological relevance and calculated the enrichment score for each subcategory.

**Whole genome sequencing analysis**

We used FastQC (Andrews, 2010) to obtain the quality control result from the raw FASTQ files of each sample, and we adopted BWA-MEM v0.7.17-r1188 (Li, 2013) to align the FASTQ files against the human reference genome hg38 downloaded from the GATK website (https://console.cloud.google.com/storage/browser/genomics-public-data/resources/broad/hg38/v0). The outputted SAM format files were converted to BAM format files using Samtools v1.6 (Li et al., 2009). We used sambamba v0.8.2 (Tarasov et al., 2015) to sort the BAM files and remove duplicates from them. We used Samtools-stats to calculate the average sequencing depth and the coverage rate of each sample.

Then we adopted the Mutect2 function of GATK v4.2.5.0 (McKenna et al., 2010) to call somatic mutations from the BAM files. Specifically, we used the genomic DNA isolated from the psoriasis lesion and contralateral control skin and employed the variation information from the 1000 Genomes project as the panel-of-normals. We filtered the somatic mutation information using the FilterMutectCalls function of GATK and default parameters. Finally, we used ANNOVAR (Wang et al., 2010) to annotate these filtered somatic mutations with the refGene database and the knownGene database (https://hgdownload.soe.ucsc.edu/goldenPath/hg38/bigZips/genes/).

**Cell Culture**

All the cell lines were cultured at 37˚C in a cell incubator with 5% CO_2_. 293FT cells (Thermo Fisher Scientific, Cat #R70007) used for virus package were maintained in DMEM (GIBCO) medium supplemented with 10% (v/v) FBS (GIBCO), 1% (v/v) Pen-strep/L-glut (Lonza), 1% (v/v) 100 mM Sodium Pyruvate (Lonza), 1% (v/v) 7.5% sodium bicarbonate (Lonza). Immortalized human KCs (HaCaT) were cultured in DMEM(GIBCO), supplemented with 10% (v/v) FBS (GIBCO), 1% (v/v) Pen-strep/L-glut (Lonza).

**Lentivirus infection**

For the overexpression of *HRAS*, *HRAS^G12S^*, full-length *HRAS* cDNA was amplified from human cDNA and then subcloned into pLKO.1 vector. Transfection of 293FT cells was carried out with pLKO.1 and helper plasmids pMD2.G and psPAX2. Viral supernatant was collected 46–48 h after transfection, filtered through a 0.45-μm filter.

The day before the infection, seed HaCaT cells at a density of 300K/well of 6-well plates. Mix 500 µL polybrene (10 mg/mL stock in PBS) with 4.5 mL FBS. Add viral supernatant of desired volume media to 1.8 mL. Add 200 µL of polybrene/serum mix per well (for a total volume of 2 mL). Incubate 30 minutes at 37°C/7.5% CO_2_. Spin plate at 1100 x g for 30 minutes at 37°C. Remove viral media and replace with culture media. Cells were cultured under serum starved condition overnight followed by 30 min stimulation with 20% serum before protein extraction.

**Western blot**

Denatured protein extracted from HaCaT cells was loaded on a 10% SDS-PAGE gel, electrophoresed, and then transferred onto a PVDF membrane. PVDF membranes were blocked with 5% fat free milk, and the membranes were then probed with p44/42 MAP kinase (ERK1/2) (CST), phospho-p44/42 MAP kinase (ERK1/2) (Thr202/Tyr204) (CST), Akt (CST), phospho-Akt (Ser473) (CST) and anti-Actin-HRP (MBL) at 4˚C overnight. Blots were then incubated with secondary antibody. Peroxidase activity on the membrane was visualized on X-ray film using the ECL western blotting detection system.

**Quantification and statistical analysis**

Data are presented as the mean value ± the standard deviation (SD). Western blot band intensity was measured by Image J and quantified in GraphPad Prism.

**Data availability**

Further sequencing data analysis information should be directed to and will be fulfilled by Dr. Bin Li (libin@nibs.ac.cn); all the other further information should be directed to and will be fulfilled by Dr. Ting Chen (chenting@nibs.ac.cn) or Dr. Wenhui Wang (wwh0608@126.com).

**References**

Andrews, S. (2010). FastQC: a quality control tool for high throughput sequence data. Babraham Bioinformatics, Babraham Institute, Cambridge, United Kingdom.

Hao, Y., Hao, S., Andersen-Nissen, E., Mauck, W.M., 3rd, Zheng, S., Butler, A., Lee, M.J., Wilk, A.J., Darby, C., Zager, M., et al. (2021). Integrated analysis of multimodal single-cell data. Cell *184*, 3573-3587.e3529. 10.1016/j.cell.2021.04.048.

Li, H. (2013). Aligning sequence reads, clone sequences and assembly contigs with BWA-MEM. arXiv preprint arXiv:1303.3997.

Li, H., Handsaker, B., Wysoker, A., Fennell, T., Ruan, J., Homer, N., Marth, G., Abecasis, G., and Durbin, R. (2009). The sequence alignment/map format and SAMtools. Bioinformatics *25*, 2078-2079.

McGinnis, C.S., Murrow, L.M., and Gartner, Z.J. (2019). DoubletFinder: Doublet Detection in Single-Cell RNA Sequencing Data Using Artificial Nearest Neighbors. Cell Syst *8*, 329-337.e324. 10.1016/j.cels.2019.03.003.

McKenna, A., Hanna, M., Banks, E., Sivachenko, A., Cibulskis, K., Kernytsky, A., Garimella, K., Altshuler, D., Gabriel, S., and Daly, M. (2010). The Genome Analysis Toolkit: a MapReduce framework for analyzing next-generation DNA sequencing data. Genome research *20*, 1297-1303.

Tarasov, A., Vilella, A.J., Cuppen, E., Nijman, I.J., and Prins, P. (2015). Sambamba: fast processing of NGS alignment formats. Bioinformatics *31*, 2032-2034.

Wang, K., Li, M., and Hakonarson, H. (2010). ANNOVAR: functional annotation of genetic variants from high-throughput sequencing data. Nucleic acids research *38*, e164-e164.

Wu, T., Hu, E., Xu, S., Chen, M., Guo, P., Dai, Z., Feng, T., Zhou, L., Tang, W., Zhan, L., et al. (2021). clusterProfiler 4.0: A universal enrichment tool for interpreting omics data. Innovation (Camb) *2*, 100141. 10.1016/j.xinn.2021.100141.
